# Distinct mouse models correspond to distinct AD molecular subtypes

**DOI:** 10.1002/alz.087565

**Published:** 2025-01-03

**Authors:** Ravi S Pandey, Gregory W Carter, Gareth R Howell, Michael Sasner, Kevin P Kotredes, Adrian L Oblak, Bruce T. Lamb

**Affiliations:** ^1^ The Jackson Laboratory for Genomic Medicine, Farmington, CT USA; ^2^ The Jackson Laboratory, Bar Harbor, ME USA; ^3^ Stark Neurosciences Research Institute, Indiana University School of Medicine, Indianapolis, IN USA

## Abstract

**Background:**

Alzheimer’s disease (AD) is a complex, multifactorial pathology with high heterogeneity in biological alterations. Our understanding of cellular and molecular mechanisms from disease risk variants to various phenotypes is still limited. Mouse models of AD serve as indispensable platforms for comprehensively characterizing AD pathology, disease progression, and biological mechanisms. However, selection of the right model in preclinical research and translation of findings to clinical populations are intricate processes that require identification of pathophysiological resemblance between model organisms and humans. Many existing clinical trials that showed promising efficacy in one particular mouse model later do not align with human trial results, assuming that study had consisted of a heterogeneous group of participants, and individual animal models may only recapitulate features of a subgroup of human cases. To improve interspecies translation, it is necessary to comprehensively compare molecular signatures in mouse models with subgroup of human AD cases with distinct molecular signatures.

**Method:**

We performed transcriptomic and proteomics analysis on whole brain samples from mouse models carrying LOAD risk variants. To assess the human disease relevance of LOAD risk variants in mice, we determined the extent to which changes due to genetic perturbations in mice matched those observed in human AD subtypes and disease stages of AD in the ROS/MAP, Mayo and Mount Sinai Brain Bank cohorts. Genesets within these disease subtypes are highly co‐expressed and represent specific molecular pathways.

**Result:**

We have identified that distinct mouse models match to distinct human AD subtypes in age‐dependent manner. Specifically, mouse models carrying human AD risk variants such as Abca7*A1527G showed strong correlation with inflammatory AD subtypes, while mouse models carrying risk variant such as Plcg2*M28L exhibited transcriptomics changes similar to non‐inflammatory AD subtypes.

**Conclusion:**

In this study, we highlighted that mouse model of AD may match to a particular subset of human AD subtypes but not all subtypes simultaneously, and that risk for these subtypes may be influenced by distinct AD genetic factors. Additional work toward validating and better understanding the role of each subtype key regulator in its matching mouse model will provide great value and have a great impact on future studies of AD.

